# Neurofolicullar hamartoma presenting as a rare adnexal neoplasm in association with basal cell carcinoma

**DOI:** 10.22088/cjim.10.1.107

**Published:** 2019

**Authors:** Fatemeh Montazer, Alireza Sanei Motlagh

**Affiliations:** 1 Department of Pathology, Gastrointestinal Cancer Research Center, Imam Hospital, Mazandaran University of Medical Sciences, Sari, Iran; 2 Department of Pathology, Iran University of Medical Sciences, Tehran, Iran; 3 Deputy of medicine, Mazandaran University of Medical Sciences, Sari, Iran

**Keywords:** Skin tumor, neurofollicular hamartoma, Basal cell carcinoma, S-100 protein, immunohistochemistry

## Abstract

**Background:**

Neurofollicular hamartoma (NFH) is characterized histopathologically by fascicles of spindle cells that laterally delimited by hyperplastic folliculosebaceous units. It usually appears on face, near the nose or nasolabial fold. It does not manifest true neural differentiation and recently the term spindle cell predominant trichodiscoma (SCPT) has been used instead.

**Case Presentation:**

We present a case of a 40-year-old male with co-incidence of NFH and basal cell carcinoma (BCC) that the mesenchymal components of NFH were similar to SCPT but these components highly expressed S-100 protein. We also discuss about the histological aspect of the neoplasia in this report and consider the findings of other reports in association with classification of NFH by means of cellular markers and morphological resemblance to other skin hamartomas.

**Conclusion:**

Neurofollicular hamartoma is a rare benign tumor that thought to represent the cellular end of a morphological spectrum with trichodiscoma. The morphological features and expression of S100 protein in neural element helped us to achieve the diagnosis of neurofollicular hamartoma. However, variable reports of S-100 protein expression in NFH are available and further studies are needed to determine the classification of this tumor.

Neurofollicular hamartoma (NFH) is a rare neoplasm that was first described by Barr and Goodman in 1989. It is characterized histopathologically by fascicles of spindle cells that are haphazardly arranged and laterally delimited by hyperplastic folliculosebaceous units. This neoplasm exhibits both epithelial and stromal components. The epithelial component consists of distorted hyperplastic pilosebaceous units, while the stroma reveals neuroid differentiation. It usually appears on face, especially near the nose or nasolabial fold (1, 2). Despite its name, it does not manifest true neural differentiation and recently the term spindle cell predominant trichodiscoma (SCPT) has been used instead. Adults are affected in the fourth or the fifth decades of life and there is no gender preponderance (3). Due to similarities of NFH to neural elements and high resemblance to trichodiscoma, and considering the observations in some reports, the precise classification of this neoplasm is controversial. We report a rare case of a middle-aged patient with two facial lesions who was primarily diagnosed with basal cell carcinoma but further pathological study revealed the co-incidence of neurofollicular hamartoma. We also discussed about the morphology and histology of this tumor and its similarity to other skin hamartomas.

## Case Presentation

**Figure 1:**
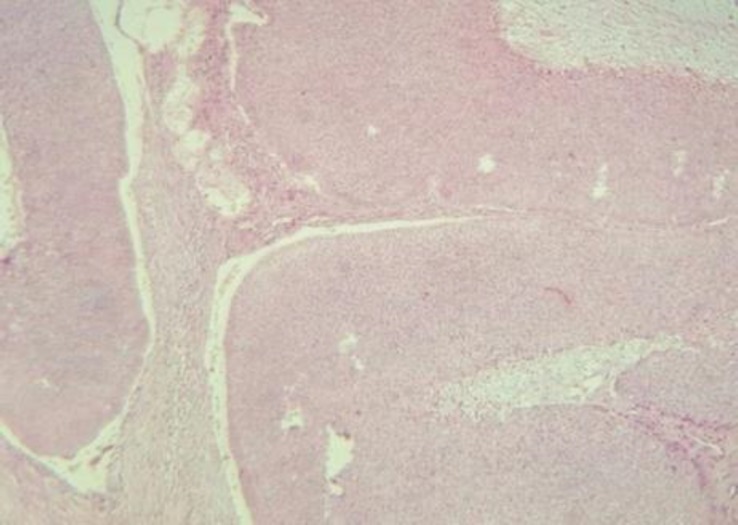
Hematoxylin and eosin (H&E) staining of the larger lesion showing basal cell carcinoma (BCC), nodular type

A 40-year-old male presented with the appearance of skin lesions on his face during the past two months. Past medical history of diabetes mellitus was noted for him without any genetic disorder. On clinical examination, two skin-colored dome shaped firm masses were noted with each size of 10×5mm and 5×5mm, respectively. The larger lesion showed surface ulcerations. With suspicion of basal cell carcinoma (BCC) for both lesions, excisional biopsy was performed and sent for pathological study including hematoxylin and eosin (H&E) and immunohistochemical (IHC) staining for S-100, neuron specific enolase (NSE), smooth muscle actin (SMA), and desmin markers. Histopathological results confirmed the BCC, nodular type, for the larger one (figure 1).

**Figure 2:**
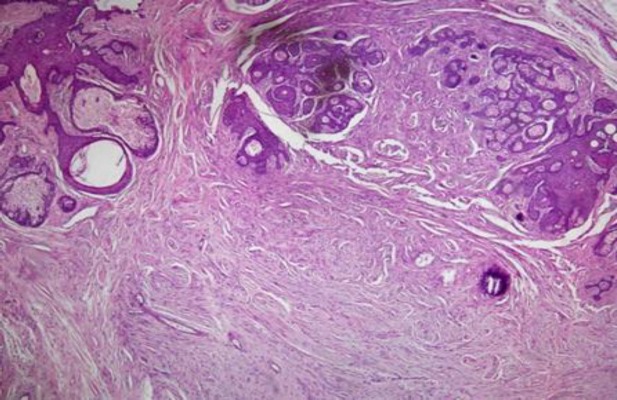
Distorted and hyperplastic pilosebaceous units with prominent sebaceous glands. H&E staining of epithelial and mesenchymal components

H&E stained sections of smaller lesion showed epidermis with unremarkable changes. The dermis displayed a well circumscribed, non-encapsulated lesion composed of epithelial and mesenchymal components. The epithelial component consist of distorted and hyperplastic pilosebaceous units with prominent sebaceous glands (fig 2). 

**Figure 3:**
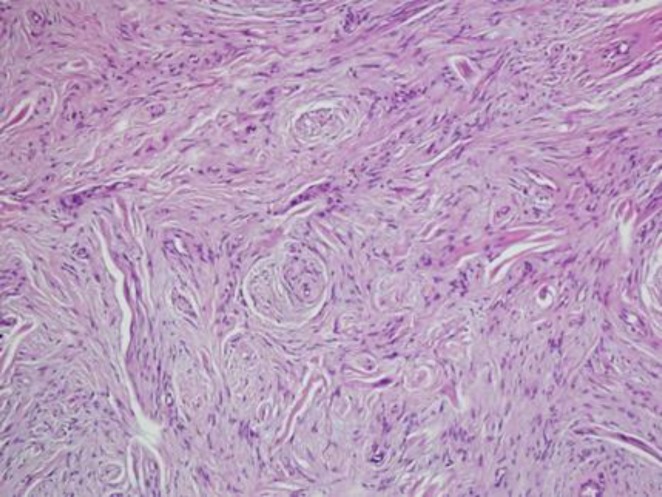
Myxoid and fibrillary appearance containing elongated and wavy spindle cells (H&E staining)

The mesenchymal component mainly showed myxoid and fibrillary appearance containing elongated and wavy spindle cells arranged in fascicles resembling neurofibroma (figure 3). 

**Figure 4:**
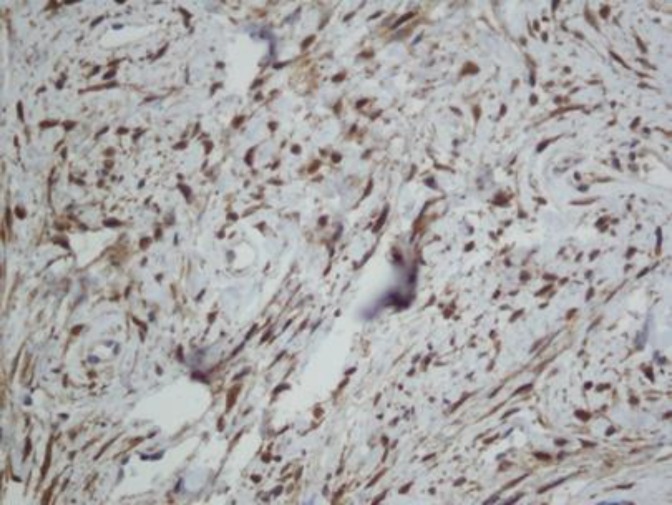
Immunohistochemical staining, fibrillary mesenchymal components express S-100 marker

**Figure 5:**
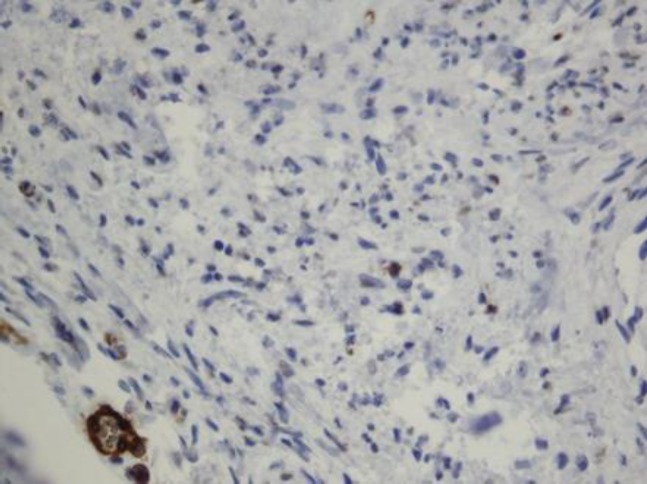
NSE and SMA, and desmin markers were negative in IHC staining of mesenchymal components

Further immunohistochemical study for confirmation of neural mesenchymal stroma was done. Fibrillary mesenchymal componenets express S*-*100 marker (figure 4), while neuron specific enolase, smooth muscle actin, and desmin were negative (figure 5). Based on the results of H&E staining and IHC, the diagnosis of neurofollicular hamartoma was confirmed. Neurofollicular hamartoma itself is a benign tumor and treatment was achieved by local excision. The BCC lesion was demarcated and small in size which was surgically removed without further topical treatment or radiotherapy. 

## Discussion

Neurofollicular hamartoma as a rare benign tumor is thought to represent the cellular end of a morphological spectrum with trichodiscoma. The clinical differential diagnosis includes fibrous papule, basal cell carcinoma, and dermal nevus (3). NFH is the tumor with follicular differentiation that expresses diffuse CD34 stromal positivity, Bcl-2 on outermost basal cells, and CD10 on perifollicular and peritumoral stroma. It shares these features with basaloid follicular hamartomas (BFH) and vellus hair hamartoma (VHH) which have the same follicular growth pattern (4). A case report of neurofollicular hamartoma showed that these lesions could express strong and diffuse pattern of S-100 protein on spindled cells. Scattered positivity of spindle cells for monoclonal neuron specific enolase and synaptophysin was also noted (5). The pathologic study results in our patient demonstrated that the lesion had myxoid and fibrillary components with wavy spindle cells just as SCPT, but these components highly expressed S-100 marker along with it. Kutzner *et al. *stated that NFH is CD34-positive cellular trichodiscoma that contain fascicles of fibrocystic spindle cells. They noted that mesenchymal component of NFH consists of wavy nuclei with focal S-100 positivity without any sign of neural differentiation. They used the term spindle cell predominant trichodiscoma (SCPT) instead of neurofollicular hamartoma to describe precise morphological the entity. Differential diagnosis of NFH should include all fairly circumscribed superﬁcial cellular lesions composed of CD34 positive spindle cells. Despite clinicopathological resemblance of angiofibroma to NHF, angiofibroma shows a dense collagenous stroma with a diffuse scatter of single cells in conjunction with more capillary vessels (6). Kacerovska et al. reported a case of SCPT with a focal palisaded arrangement of stromal cell just like those seen in schwannoma, in which the stromal cells were positive for CD34 and negative for S-100 protein. The similarity of SCPT to peripheral nerve neoplasms was mentioned by illustrations (7). 

Basal cell carcinoma (BCC) is the most common cutaneous malignant neoplasm. It has slow growth pattern and rarely metastasize but a small proportion (less than 0.5%) of this tumor is locally aggressive. Nests of uniform basaloid cells within the dermis that are often separated from the adjacent stroma by the thin clefts are apparent in H&E stained sections (8). The lesions in this patient were small with clear margins and they surgically removed with consideration of cosmetics. In this report (to our knowledge), we describe the first co-incidence of BCC and neurofollicular hamartoma. The morphological features of smaller lesion and also expression of S100 protein in neural element helped us to achieve the diagnosis of neurofollicular hamartoma. Based on variable reports of S-100 protein expression in NFH, as well as its similarity to peripheral nerve tumors, further studies are needed to determine the classification and differentiation of NFH.
